# Microvolt T-wave alternans and autonomic nervous system parameters can be helpful in the identification of low-arrhythmic risk patients with ischemic left ventricular systolic dysfunction

**DOI:** 10.1371/journal.pone.0196812

**Published:** 2018-05-03

**Authors:** Ludmiła Daniłowicz-Szymanowicz, Damian Kaufmann, Katarzyna Rozwadowska, Maciej Kempa, Ewa Lewicka, Grzegorz Raczak

**Affiliations:** Department of Cardiology and Electrotherapy, Medical University of Gdansk, Gdansk, Poland; University of Minnesota, UNITED STATES

## Abstract

**Introduction:**

The role of implantable cardioverter-defibrillator (ICD) placement in the primary prevention of sudden cardiac death (SCD) in all consecutive patients with left ventricular ejection fraction (LVEF) ≤ 35% is still a matter of hot debate due to the fact that the population of these patients is highly heterogeneous in terms of the SCD risk. Nevertheless, reduced LVEF is still the only established criterion during qualification of patients for ICD implantation in the primary prevention of SCD, therefore identification of persons with particularly high risk among patients with LVEF ≤35% is currently of lesser importance. More important seems to be the selection of individuals with relatively low risk of SCD in whom ICD implantation can be safely postponed. **The aim of the study** was to determine whether well-known, non-invasive parameters, such as microvolt T-wave alternans (MTWA), baroreflex sensitivity (BRS) and short-term heart rate variability (HRV), can be helpful in the identification of low-arrhythmic risk patients with **ischemic** left ventricular systolic dysfunction.

**Methods:**

In 141 patients with coronary artery disease and LVEF ≤ 35%, MTWA testing, as well as BRS and short-term HRV parameters, were analysed. During 34 ± 13 months of follow-up 37 patients had arrhythmic episode (EVENT): SCD, non-fatal sustained ventricular arrhythmia (ventricular tachycardia [VT] or ventricular fibrillation [VF]), or adequate high-voltage ICD intervention (shock) due to a rapid ventricular arrhythmia ≥200/min. LVEF, non-negative MTWA (MTWA_non-neg), BRS and low frequency power in normalized units (LFnu) turned out to be associated with the incidence of EVENT in univariate Cox analysis. The cut-off values for BRS and LFnu that most accurately distinguished between patients with and without EVENT were 3 ms/mmHg and 23, respectively. The only variable that provided 100% negative predictive value (NPV) for EVENT was negative MTWA result (MTWA_neg), but solely for initial 12 months of the follow-up; the NPVs for other potential predictors of the EVENT were lower. The cut-off values for BRS and LFnu that provide 100% NPV for EVENT during 12 and 24 months were higher: 6.0 ms/mmHg and 73 respectively, but the gain in the NPV occurred at an expense of the number of identified patients. However, the number of identified non-risk patients turned out to be higher when the predictive model included MTWA_neg and the lower cut-off values for ANS parameters: 100% NPV for 12 and 24 months of follow-up was obtained for combination MTWA_neg and BRS ≥ 3 ms/mmHg, for combination MTWA_neg and LFnu ≥ 23 100% NPV was obtained for 12 months.

**Conclusion:**

Well-known, non-invasive parameters, such as MTWA, BRS and short-term HRV indices may be helpful in the identification of individuals with a relatively low risk of malignant ventricular arrhythmias among patients with ischemic left ventricular systolic dysfunction; in such persons, implantation of ICD could be safely postponed.

## Introduction

Sudden cardiac death (SCD) constitutes a significant problem in contemporary cardiology, and implementation of implantable cardioverters-defibrillators (ICD) to clinical practice was a breakthrough in the prevention of this condition [1]. While the necessity for ICD implantation in persons with a history of cardiac arrest due to malignant ventricular arrhythmia (ventricular tachycardia [VT] or ventricular fibrillation [VF]) raises no controversies, the role of these devices in the primary prevention of SCD in patients with left ventricular systolic dysfunction, i.e. with left ventricular ejection fraction (LVEF) ≤35%, is still a matter of hot debate [2–4]. This results from the fact that the population of patients with LVEF ≤35% is highly heterogeneous in terms of the SCD risk, which contributes to low sensitivity and specificity of left ventricular ejection fraction as a predictor of VT/VF [5]. Up to 76% and up to 79% of participants of the two largest studies analysing the role of ICD in left ventricular systolic dysfunction, MADIT II and SCD-HeFT, respectively, did not benefit from the implantation of these devices during a 21- and 45.5-month-long follow-up [6, 7]. Furthermore, it needs to be stressed that in everyday clinical practice, the ICDs are frequently implanted in older patients who typically present with more comorbidities than the participants of clinical trials; consequently, the actual proportion of patients who do not benefit from this type of intervention may be even higher. On the other hand, reduced LVEF is a risk factor for both SCD and non-sudden cardiac death [8–10], and the likelihood of the latter is not mitigated by the ICD implantation.

Nevertheless, reduced LVEF is still the only established criterion during qualification of patients for the primary prevention of SCD with ICD therapy [1]. Consequently, identification of persons with particularly high risk of SCD among patients with LVEF ≤35% is currently of lesser importance. More important seems to be the selection of individuals with relatively low risk of VT/VF in whom ICD implantation can be safely postponed. Such approach seems to be helpful in optimization waiting lines for ICD implantation, by postponing the procedure in patients with relatively low likelihood of SCD and prioritization those at increased risk. This might contribute to a decrease SCD numbers among patients awaiting ICD implantation, which seems to be particularly important in the case of countries with tight healthcare budgets. Moreover, selection of individuals with relatively low risk of VT/VF seems to be particularly justified in persons with temporary contraindications to ICD implantation, for example with infective endocarditis or increased risk of infectious complications [11].

The aim of the study was to determine whether well-known, non-invasive parameters, such as microvolt T-wave alternans (MTWA), baroreflex sensitivity (BRS) and short-term heart rate variability (HRV), can be helpful in the identification of low-arrhythmic risk patients with ischemic left ventricular systolic dysfunction, in whom the primary prevention of SCD with ICD therapy can be safely postponed.

## Materials and methods

The protocol of the study was approved by the Local Ethics Committee at the Medical University of Gdansk, and written informed consent was obtained from all the participants.

### Patient selection

Between December 2012 and May 2014, consecutive patients with coronary artery disease (CAD) and left ventricular systolic dysfunction (LVEF ≤35%), qualified for the ICD implantation within the framework of the primary prevention of SCD, and managed in accordance with the current recommendations, were prospectively enrolled to the study. The definition of CAD was based on data from the patient's medical records: history of previous myocardial infarction, and/or previous revascularization treatment, and/or at least 50% stenosis in coronary arteries found during coronarography performed before the enrollment. The exclusion criteria were: age below 18 years, history of prior sustained ventricular arrhythmia or cardiac arrest, presence of permanent atrial fibrillation/flutter, permanent second- or third- degree atrioventricular block, implantation of a pacemaker, New York Heart Association (NYHA) functional class IV heart failure, clinical features of coronary instability at enrolment time, a coronary angioplasty or / and surgery by-pass during the 3 months prior to the study, incomplete coronary revascularization status (scheduled control coronarography, coronary angioplasty or surgery by-pass), the inability to exercise on a treadmill, poor general condition (concomitant terminal disease), and non-cardiologic comorbidities with potential unfavourable effect on survival.

The protocol of the baseline visit included medical history, taking medications, physical examination and a 12-lead electrocardiogram. The ultimate decision to implant or not implant an ICD in a given patient was left at the discretion of his/her physician in charge. The study was conducted as a part of the larger research project on the risk stratification in life-threatening ventricular arrhythmias.

### MTWA testing

All patients were instructed to continue their current pharmacotherapy, including beta-blockers. After appropriate preparation of patient’s skin to minimise the risk of artefacts (cleansing with an abrasive paper), the electrodes were placed in three orthogonal Frank leads (X, Y and Z; High-Res high-resolution electrodes, *Cambridge Heart—Spacelabs Healthcare*, *Snoqualmie*, *WA*, *USA*), as well as in 12 standard leads. The exercise test was performed on a treadmill (*Delmar Reynolds*), in line with the protocol for MTWA testing, i.e. with a gradual increment in heart rate, first to 100–110 beats per minute (bpm) and then to 110–120 bpm (for at least 2 minutes). MTWA was analysed using the spectral method *(Cambridge Heart—Spacelabs Healthcare*, *Snoqualmie*, *WA*, *USA)*. Aside from the computer-guided analysis, the results were also evaluated by the physician who supervised the test. The result of the test was classified as negative (MTWA_neg), positive or indeterminate, according to generally accepted criteria [12]. Since in patients with left ventricular systolic dysfunction, either positive or indeterminate result of the test is associated with poor prognosis [12], all non-negative results (MTWA_non-neg) were considered abnormal and analysed jointly.

### ANS parameters

The ANS tests were performed between 08:00 am and 1:00 pm. Patients were instructed to continue their current pharmacotherapy, but refrain from eating for at least 4 hours, and from smoking cigarettes and drinking coffee for at least 12 hours prior to the examination. The recordings were obtained in a quiet room, with the patient relaxed in the supine position with head elevated by 30°. After a 15-minute stabilization in the supine position, resting ECG (*Mingograf 720C*) and *beat-to-beat* non-invasive arterial blood pressure (*Finapres 2300*, *Ohmeda*) were recorded continuously for 10 minutes. The signals were acquired with a PC workstation, processed with a dedicated software [13] and analysed according to the protocol described elsewhere [14, 15]. The data on RR interval (1-ms resolution) and systolic arterial pressure (SAP) were acquired automatically. BRS (ms/mmHg) was computed by spectral analysis as the average value of the transfer function modulus (Blackman-Tukey method, 0.03 Hz-bandwidth Parzen window) between SAP and RR interval time series in the low frequency (LF, 0.04–0.15 Hz) band, independently from coherence values *(whole band average)* [14]–that means not only values having magnitudesquared coherence of ≥ 0.5. This method is clearly described by Pinna et al. [14]. Furthermore, routine HRV indices: total power (TP, ms^2^), relative spectral power in LF (LFnu, expressed in normalized units) and LF to high frequency (HF, 0.14–0.4 Hz) ratio (LF/HF) were analysed. Also the following time-domain HRV parameters were calculated based on the RR data: the standard deviation of normal-to-normal RR intervals (SDNN), the square root of the mean of squared differences between successive intervals (RMSSD) and the percentage of adjacent RR intervals differing by more than 50 ms (pNN50) [16]. Finally, mean heart period (HP, ms) value was recorded and subjected to the analysis.

### Follow-up

The patients were followed-up at the university outpatient clinic. The first visit was scheduled within 3 months of enrolment; subsequently, the patients were followed-up every 6 months, or earlier if clinically required. During each visit, patient’s clinical status was evaluated and all adverse events were recorded, if any.

The primary endpoint (EVENT) of this study was SCD, non-fatal sustained ventricular arrhythmia (VT or VF), or adequate high-voltage ICD interventions (shocks) due to a rapid ventricular arrhythmia ≥200/min. The relevance of the intervention was verified based on the analysis of the electrograms stored in the ICD’s memory. Due to proper programming of the ICDs, described above, we were able to exclude all potential non-persistent episodes from the analysis. Patients with more than one VT or ICD discharge were classified as reaching the primary endpoint after the first such episode. SCD was diagnosed according to the widely accepted definition, as an unexpected death due to cardiac causes occurring within 60 minutes of symptom onset and preceded by a loss of consciousness, or as an unexpected death without witnesses occurring in a person who did not report any ailments within the last 24 hours. All deaths were verified against medical documentation of the patient and/or death certificate information.

### Statistical analysis

The minimum sample size was estimated using the following mathematical formula: *n* = (1.96/0.2)^2^ = 97 (95% confidence interval (CI) would not exceed 20% [the error of the estimation would not exceed 10%]). For safety, the sample size was set at 140 (the accuracy was improved and the error value was 8.5%). Due to the lack of normal distribution, quantitative data for EVENT_(+) and EVENT_(−) groups were compared with Mann-Whitney test, and the qualitative data with chi-square test or Yates’ chi-square test (depending on the sample size). The accuracy of ANS indices as potential predictors of the study endpoint was determined based on the area (AUC) under the receiver-operating characteristic (ROC) curve. To identify the cut-off values for the study parameters that most accurately distinguished between EVENT_(+) and EVENT_(−) groups, we optimized the procedure of ROC curve analysis; namely, we selected the values with the maximum sum of positive (PPV) and negative predictive value (NPV): PPV + NPV. A given cut-off value was considered to be associated with low risk of the event if the AUC under its ROC curve was greater than 95%. Since ROC curve is based on the sensitivity and specificity of a given predictor, we first determined these two parameters, then PPV and NPV, and finally, the cut-off value that most accurately distinguished between the study groups. Then, prognostic value of dichotomous variables identified based on the cut-off values was verified using univariate and multivariate Cox proportional hazard models, with the study endpoint as the outcome variable. To avoid overfitting, we restricted the number of predictors in the final multivariate model to *m*/10, where *m* is the number of observed events [17]. Therefore, the number of variables in the clinical model was *m*/10–1. The probabilities of reaching the primary endpoint over time, stratified according to the MTWA result and the cut-off values for ANS parameters, were estimated using Kaplan-Meier method and compared with log-rank test. The sensitivity, specificity, PPV and NPV of MTWA as a predictor of the study endpoint were presented along with their 95% confidence intervals (95% CIs). The results were considered statistically significant for p-values ≤0.05. The statistical analysis was carried out with STATISTICA 9.0 (*StatSoft*, *Tulsa OK*, *USA*) package and R 2.15.2 environment.

## Results

### Clinical characteristics of the studied patients

All participants of the study were recruited as outpatients (S1 Table). Clinical characteristics of 141 patients enrolled in this study are listed in Table 1; the vast majority (90%) of them were males, with mean age of 64 years and mean LVEF of 30%. The result of MTWA test was negative in 42 patients (30%), positive in 69 (49%) and indeterminate in 30 (21%).

**Table 1:** Clinical and demographic characteristics of the study group and comparison between the EVENT_(+) and EVENT_(−) groups.

	All N = 141	EVENT_(+) N = 37	EVENT_(−) N = 104	p*
Age [years]	64 (58–72)	65 (59–72)	64 (58–72)	0.233
Males, n (%)	127 (90)	32 (86)	95 (91)	0.522
MI history n (%)	127 (90)	31 (84)	96 (92)	0.197
Revascularization, n (%)	127 (90)	31 (84)	96 (92)	0.197
LVEF (%)	30 (25–32)	28 (23–32)	32 (26–35)	**<0.007**
NYHA class				0.282
- NYHA I, n (%)	23 (16)	3 (8)	20 (19)	
- NYHA II, n (%)	89 (63)	25 (68)	64 (62)	
- NYHA III, n (%)	29 (21)	9 (24)	20 (19)	
QRS≥120 ms, n (%)	86 (61)	27 (73)	59 (57)	0.116
VPCs>10/godz., n (%)	65 (46)	18 (49)	47 (45)	0.848
nsVT, n (%)	52 (37)	17 (46)	35 (33)	0.234
MTWA_non-neg, n (%)	99 (70)	35 (95)	64 (62)	**<0.001**
- beta-adrenolytics, n (%)	135 (96)	36 (97)	99 (95)	1.000
- ACE-inhibitor or ARB, n (%)	132 (94)	34 (92)	98 (94)	0.698
- spironolactone, eplerenone, n(%)	76 (54)	18 (49)	58 (56)	0.565
- aspirin, n (%)	141 (100)	37 (100)	104 (100)	1.000
- amiodarone, n (%)	16(11)	3 (8)	13 (13)	0.561
- statins, n (%)	141 (100)	37 (100)	104 (100)	1.000
- digoxin, n (%)	6 (4)	3 (8)	3 (3)	0.185
-arterial hypertension, n (%)	93 (65)	19 (51)	74 (71)	**<0.043**
- diabetes, n (%)	41 (29)	12 (32)	29 (28)	0.674
Renal function:				0.205
GFR>60 ml/min, n (%)	104 (74)	30 (81)	74 (71)	
GFR 30–59 ml/min, n (%)	32 (23)	5 (14)	27 (26)	
GFR<30 ml/min, n (%)	5 (3)	2 (5)	3 (3)	
- hypercholesterolaemia, n (%)	98 (70)	27 (73)	71 (68)	0.680
- history of tobacco smoking, n (%)	102 (72)	28 (76)	74 (71)	0.673
ICD (including CRT-D)	107 (76)	29 (78)	78 (75)	0.824
CRT-D	14 (10%)	4 (11%)	10 (10%)	1.000

Abbreviations: ACE–angiotensin converting enzyme, ARB–angiotensin receptor blockers; MI–myocardial infarction; LVEF–left ventricular ejection fraction; NYHA–classification according New York Heart Association; VPCs–ventricular premature contractions, nsVT–nonsustained ventricular tachycardia; MTWA_non-neg–positive and indeterminate results for microvolt T-wave alternans; GFR–glomerular filtration ratio; ICD–implantable cardioverter-defibrillator; CRT-D–cardiac resynchronization therapy device with ICD

* p value for comparison between EVENT_(+) and EVENT_(−) groups

Mean duration of the follow-up was 34 ± 13 months (range 12–57 months); during this period, the primary endpoint was reached by 37 patients (Table 2). Patients from EVENT_(+) and EVENT_(−) groups did not differ significantly in terms of their clinical and demographic characteristics. However, the persons who reached the primary endpoint significantly more often presented with a non-negative result of MTWA test and had significantly lower LVEF (Table 1). Moreover, the study groups differed in terms of selected ANS indices: BRS, LFnu and LF/HF (Table 3).

**Table 2:** Distribution of clinical events contributing to the primary end points.

All No. of patients	141
Sudden cardiac death	10
Spontaneous sustained VT / VF	5
Appropriate ICD discharge	22

Abbreviations: VT/VF–ventricular tachycardia/ventricular fibrillation, ICD–implantable cardioverter-defibrillator.

**Table 3:** BRS and HRV parameters in patients from EVENT_(+) and EVENT_(−) groups.

	All (n = 141)	EVENT_(+) (n = 37)	EVENT_(−) (n = 104)	*p
Mean HP (ms)	1043 (955–1150)	1044 (951–1164)	1042 (956–1143)	0.312
SDNN (ms)	24.80 (15.50–36.60)	21.30 (12.80–39.65)	25.90 (17.80–34.58)	0.192
RMSSD (ms)	16.40 (10.04–29.10)	12.80 (9.90–34.15)	16.6 (10.55–27.45)	0.391
pNN50 (%)	0.54 (0–7.15)	0.05 (0–11.85)	0.61 (0–6.17)	0.463
TP (ms^2^)	421.20 (177.5–1017)	341.00 (136.50–1080.00)	503.25 (191.03–1006)	0.226
LFnu	48.90 (26.40–70.90)	31.30 (14.75–63.15)	56.00 (29.03–74.10)	**<0.033**
LF/HF	0.96 (0.36–2.44)	0.46 (0.17–1.72)	1.28 (0.41–2.86)	**<0.036**
BRS (ms/mmHg)	4.42 (2.36–6.76)	2.62 (2.05–3.63)	5.01 (2.57–9.75)	**<0.001**

Abbreviations: HP–heart period; SDNN–standard deviation of the average R-R intervals of the sinus rhythm; RMSSD–square root of the mean squared difference of successive R-R intervals; pNN50 –proportion of successive R-R intervals that differ by more than 50 ms; TP–total power; LFnu–spectral power in low-frequency range (0.04–0.15 Hz) expressed in normalized units; LF/HF–LF to HF ratio; BRS–baroreflex sensitivity

* p value for comparison between EVENT_(+) and EVENT_(−) groups

### Prognostic accuracy of the study parameters

**Fig 1:**
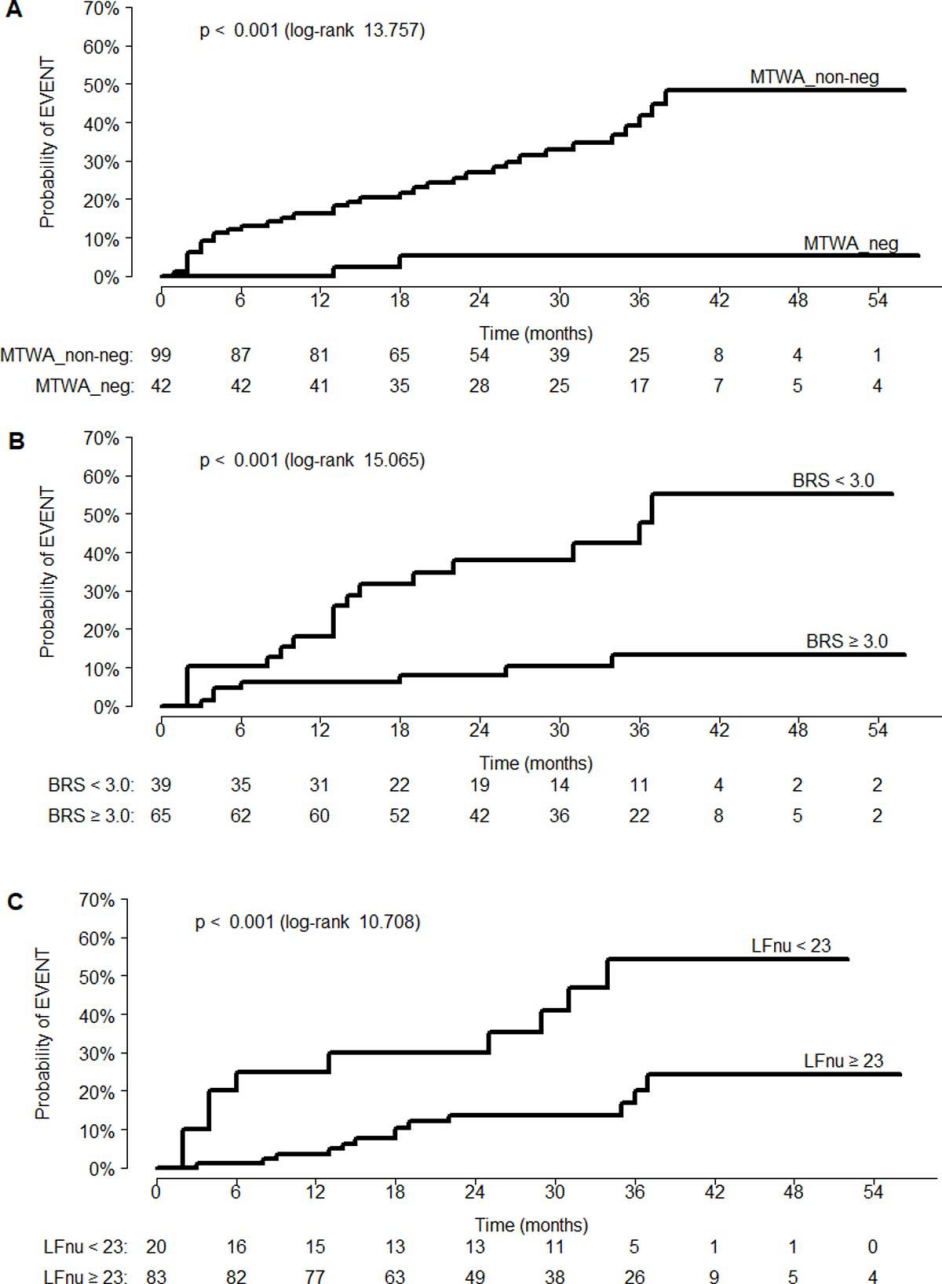
Kaplan-Meier curves illustrating probability of the EVENT during the follow-up period, stratified according to the result of MTWA test and prespecified cut-off values for BRS and LFnu. Estimated EVENT rates (95% CI) at 12 and 24 months were: **(A)** 0% (0.0–0.0) and 5.2% (0.0–12.0), respectively, for MTWA_neg, and 16.2% (8.6–23.1) and 26.9% (17.2–35.6), respectively, for MTWA_non-neg; **(B)** 6.2% (0.1–11.8) and 8.0% (1.0–14.4), respectively, for BRS≥3.0 ms/mmHg, and 18.0% (5.0–29.3) and 38.0% (19.9–52.1), respectively, for BRS<3.0 ms/mmHg; **(C)** 3.6% (0.0–7.5) and 13.7% (5.3–21.3), respectively, for LFnu≥23, and 25.0% (3.4–41.8) and 30.0% (6.7–47.5), respectively, for LFnu<23. The numbers under the figure represent the number of patients subjected to an incident risk assessment by the specified time.

ROC analysis identified LVEF as a predictor of the EVENT (AUC 65.0% [CI 54.9–75.1%]). Similar discriminatory powers were also obtained for BRS (AUC 71.9% [CI 61.7–82.1%]), LFnu (AUC 64.7% [CI 51.1–78.3%]) and LF/HF (AUC 64.4% [CI 50.8–78.0%]). LVEF, MTWA_non-neg, BRS and LFnu, but not LF/HF turned out to be associated with the incidence of the primary endpoint on univariate Cox analysis (Table 4). The cut-off values for BRS and LFnu that most accurately distinguished between patients from EVENT_(+) and EVENT_(−) groups were 3 ms/mmHg and 23, respectively; the discriminatory power of both these cut-off values as predictors of the primary endpoint was confirmed on univariate Cox analysis (Table 4), as shown on the Kaplan-Meier curve (Fig 1). When the result of MTWA test, or the cut-off value for BRS or LFnu was included in a bivariate Cox model containing LVEF, all components of the model were significant predictors of the primary endpoint (Table 4).

**Table 4:** Univariate and multivariate Cox models estimating likelihood of the EVENT during the follow-up based on the result of MTWA test, BRS and LFnu indices.

	Unadjusted		Adjusted*	
	p	Hazard ratio (95% CI)	p	Hazard ratio (95% CI)
LVEF (%)	**0.002**	0.93 (0.88–0.97)	-	-
MTWA_non-neg	**0.002**	9.13(2.19–38.00)	**0.006**	7.41 (1.76–31.18)
BRS (ms/mmHg)	**0.009**	0.82 (0.70–0.95)	**0.004**	0.76 (0.64–0.92)
BRS < 3 ms/mmHg	**0.001**	4.82 (2.00–11.65)	**0.001**	4.52 (1.87–10.96)
LFnu	**0.020**	0.98 (0.97–1.00)	**0.021**	0.98 (0.97–1.00)
LFnu < 23	**0.002**	3.63 (1.59–8.31	**0.001**	4.16 (1.80–9.62)
LF/HF	0.662	0.96 (0.81–1.15)	0.789	0.97 (0.80–1.19)

Abbreviations: CI–confidence interval; LVEF–left ventricular ejection fraction; MTWA_non-neg–positive and indeterminate results for microvolt T-wave alternans; BRS–baroreflex sensitivity; LFnu–relative spectral power in LF range, expressed in normalized units; LF/HF–LF to HF ratio

*Adjusted for LVEF

#### Negative predictive value of the study parameters

**Fig 2:**
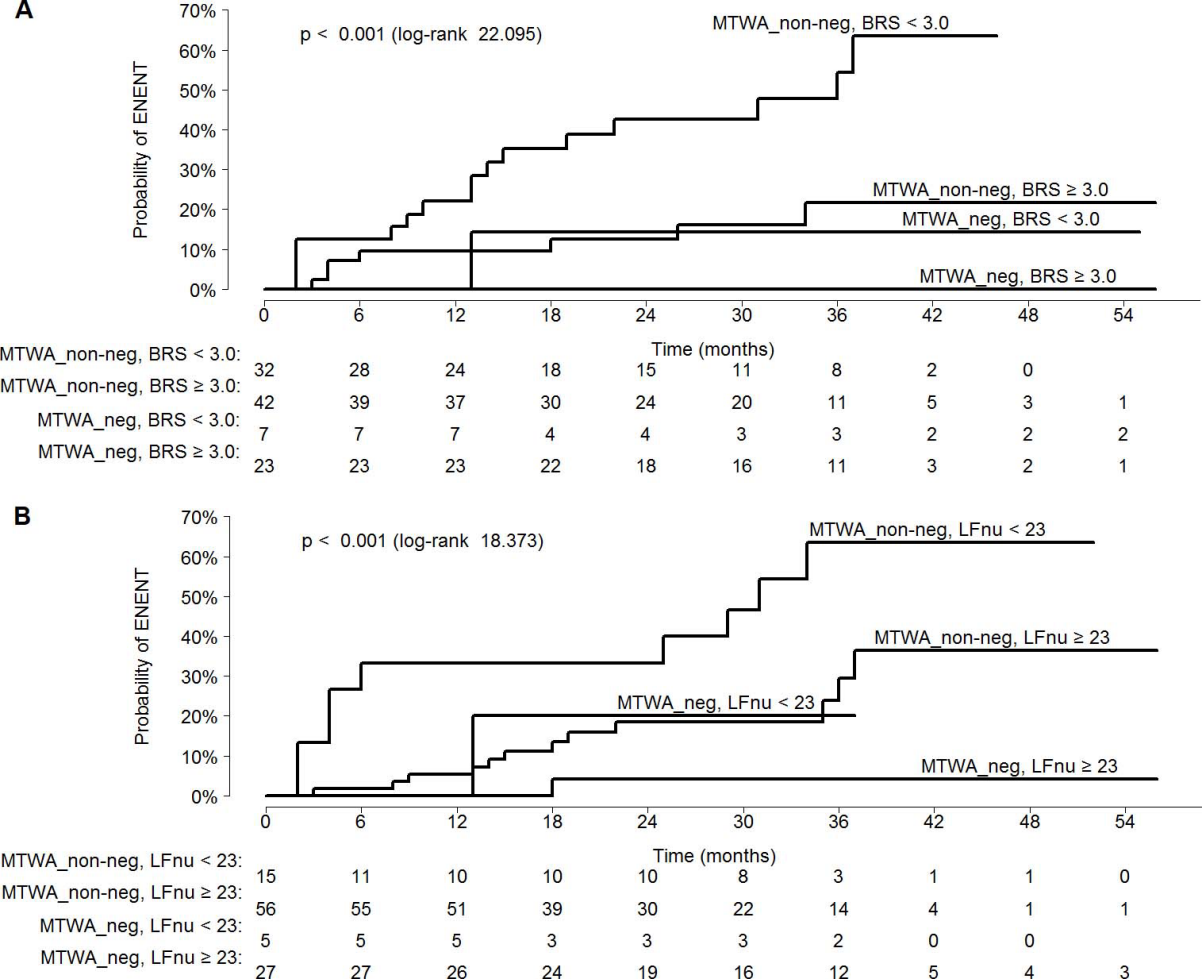
Kaplan-Meier curves illustrating probability of the EVENT during the follow-up period, stratified according to the result of MTWA test analysed jointly with prespecified cut-off values for BRS or LFnu. Estimated EVENT rates (95% CI) at 12 and 24 months were: **(A) -**0.0% (0.0–0.0) and 0.0% (0.0–0.0), respectively, for the combination of MTWA_neg and BRS≥3.0 ms/mmHg; -0.0% (0.0–0.0) and 14.3% (0.0–36.7), respectively, for the combination of MTWA_neg and BRS<3.0 ms/mmHg; -9.5% (0.2–18.0) and 12.5% (1.5–22.3), respectively, for the combination of MTWA_non-neg and BRS≥3.0 ms/mmHg; -22.0% (6.2–35.2) and 42.6% (21.8–57.9), respectively, for the combination of MTWA_non-neg and BRS<3.0 ms/mmHg **(B)** -0.0% (0.0–0.0) and 4.2% (0.0–11.8), respectively, for the combination of MTWA_neg and LFnu≥23, - 0.0% (0.0–0.0) and 20.0% (0.0–48.4), for the combination of MTWA_neg and LFnu<23; -5.4% (0.0–11.1) and 18.5% (6.6–29.0), respectively, for the combination of MTWA_non-neg and LFnu≥23; -33.3% (4.7–53.4) and 33.3% (4.7–53.4), respectively, for the combination of MTWA_non-neg and LFnu<23. The numbers under the figure represent the number of patients subjected to an incident risk assessment by the specified time.

Diagnostic accuracy of MTWA_neg and the cut-off values for BRS and LFnu as the predictors of the primary endpoint is shown in Table 5. As shown in the table, the only variable that provided 100% NPV in the prediction of the primary endpoint was MTWA_neg, but solely for initial 12 months of the follow-up; the NPVs for other potential predictors of the primary endpoint were lower. Consequently, we searched for the cut-off values for BRS and LFnu that would provide 100% NPV in the prediction of the primary endpoint during both 12- and 24-month-long follow-up, either alone or analysed jointly with MTWA_neg. While this criterion was satisfied by BRS equal to 6.0 ms/mmHg and LFnu of 73 ms^2^ (Table 5), the gain in the NPV occurred at an expense of the number of identified patients. However, the number of identified non-risk patients turned out to be higher when the predictive model included MTWA_neg and the previously identified lower cut-off values for the ANS parameters (Table 6). The likelihood of reaching the primary endpoint during the follow-up period, stratified according to the prespecified cut-off values, is presented on Fig 2.

**Table 5:** Prognostic accuracy of the study parameters (prespecified cut-off values) as the predictors of the EVENT during the follow-up.

Parameters	Follow-up period	Characteristics (%) (95% CI)		Predictive Value (%) (95% CI)	
		Sensitivity	Specificity	Positive	Negative
**MTWA_neg** (n = 42)	12 months	100.00 (80.64–100.00)	33.60 (25.92–42.26)	16.16 (10.20–24.65)	100.00 (91.62–100.00)
	24 months	89.47 (68.61–97.06)	32.79 (25.09–41.53)	17.17 (11.01–25.79)	95.24 (84.21–98.68)
**BRS≥3.0** (n = 65)	12 months	63.64 (35.38–84.83)	64.52 (54.39–73.49)	17.50 (8.75–31.95)	93.75 (85.00–97.54)
	24 months	66.67 (39.06–86.19)	65.22 (55.05–74.16)	20.00 (10.50–34.76)	93.75 (85.00–97.54)
**LFnu≥23** (n = 83)	12 months	62.50 (30.57–86.32)	84.21 (75.57–90.19)	25.00 (11.19–46.87)	96.39 (89.90–98.76)
	24 months	60.00 (31.27–83.18)	84.95 (76.30–90.82)	30.00 (14.55–51.90)	95.18 (88.25–98.11)
**BRS≥6.0** (n = 33)	12 months	100.00 (74.12–100.00)	35.48 (26.51–45.61)	15.49 (8.88–25.65)	100.00 (89.57–100.00)
	24 months	100.00 (75.75–100.00)	35.87 (26.82–46.05)	16.90 (9.94–27.26)	100.00 (89.57–100.00)
**LFnu≥73** (n = 23)	12 months	100.00 (67.56–100.00)	24.21 (16.71–33.72)	10.00 (5.15–18.51)	100.00 (85.69–100.00)
	24 months	100.00 (72.25–100.00)	24.73 (17.08–34.38)	12.50 (6.93–21.50)	100.00 (85.69–100.0

Abbreviations: MTWA_non-neg–positive and indeterminate results for microvolt T-wave alternans; BRS–baroreflex sensitivity; LFnu–relative spectral power in LF range, expressed in normalized units

**Table 6:** Prognostic accuracy of the composite measures (including prespecified cut-off values) as the predictors of the EVENT during the follow-up.

Parameters	Follow-up period	Characteristics (%) (95% CI)		Predictive Value (%) (95% CI)	
		Sensitivity	Specificity	Positive	Negative
**MTWA_neg+** **BRS≥3.0** (n = 23)	12 months	100.00 (74.12–100.00)	24.73 (17.08–34.38)	13.58 (7.76–22.70)	100.00 (85.69–100.00)
	24 months	100.00 (75.75–100.00)	25.00 (17.28–34.73)	14.81 (8.68–24.13)	100.00 (85.69–100.00)
**MTWA_neg+** **LFnu≥23** (n = 27)	12 months	100.00 (67.56–100.00)	28.42 (20.33–38.19)	10.53 (5.43–19.42)	100.00 (87.54–100.00)
	24 months	90.00 (59.58–99.49)	27.96 (19.85–37.81)	11.84 (6.36–21.00)	96.30 (81.72–99.81)
**MTWA_neg+** **BRS≥6.0** (n = 10)	12 months	100.00 (74.12–100.00)	10.75 (5.95–18.67)	11.70 (6.66–19.75)	100.00 (72.25–100.00)
	24 months	100.00 (75.75–100.00)	10.87 (6.01–18.86)	12.77 (7.46–21.00)	100.00 (72.25–100.00)
**MTWA_neg+** **LFnu≥73** (n = 6)	12 months	100.00 (67.56–100.00)	6.32 (2.93–13.10)	8.25 (4.24–15.44)	100.00 (60.97–100.00)
	24 months	100.00 (72.25–100.00)	6.45 (2.99–13.37)	10.31 (5.70–17.95)	100.00 (60.97–100.00)

Abbreviations: MTWA_non-neg–positive and indeterminate results for microvolt T-wave alternans; BRS–baroreflex sensitivity; LFnu–relative spectral power in LF range, expressed in normalized units

## Discussion

This study included patients with ischaemic left ventricular systolic dysfunction, the most common type of heart failure [18–20], associated with the highest risk of arrhythmic events [6, 21]. Patients with ischemic left ventricular systolic dysfunction are also the largest group among persons scheduled for ICD implantation within the framework of the primary prevention of SCD. Consequently, the observation that the result of MTWA test and simple non-invasive ANS parameters may be helpful in the identification of patients with the lowest risk of life-threatening ventricular arrhythmias, should be considered the principal finding of our study.

### Prognostic value of MTWA in the identification of low-arrhythmic risk patients

The prognostic value of MTWA was studied previously by many authors. However, the NPVs for MTWA documented in those studies varied considerably, from 71% [22, 23] to 100% [24–29]. Those discrepancies might be caused by a number of factors. One of them is continuation or discontinuation of beta-adrenolytic therapy at the time of the test. In most studies in which the NPV for MTWA approximated 100%, the majority of patients continued beta-blocker therapy [24–29]. Furthermore, the result of MTWA test lacked prognostic value in the studies in which beta-blocker was withdrawn 24 hours prior to the examination [30], as well as in the trials including only a small proportion of patients receiving beta-blockers [31]. Thus, we decided to continue beta-blocker therapy during MTWA test, and agents from this group were used by nearly 100% of patients enrolled in our study. Follow-up time is another factor that may influence the prognostic value of MTWA. For example, in ABCD trial, including a total of 566 patients with LVEF ≤40%, the NPV for MTWA was 95% during the first year, and then dropped off below 90% during the second [26]. A similar relationship between the duration of follow-up and the NPV for MTWA was observed in our present study (Table 5). This implies that if the decision to postpone ICD implantation is to be based solely on MTWA, this test should be periodically repeated whenever it yielded a negative result. According to many authors, the prognostic value of MTWA may also be influenced by the selection criteria for the study group. For example, a meta-analysis of studies involving individuals with a history of life-threatening ventricular arrhythmias demonstrated that the NPV for MTWA in this group was significantly lower than in other patient populations [32, 33]. Our study included carefully selected patients whose characteristics qualified them for the primary prevention of SCD by ICD therapy; during the first year of the follow-up, the NPV for MTWA in this group amounted to 100%.

### Prognostic value of ANS indices in the identification of low-arrhythmic risk patients

Likewise for MTWA, most previous studies dealing with the prognostic role of ANS indices centred around the identification of patients with the highest risk of cardiac events [34–42]. In contrast, the aim of our present study was to determine the values of BRS and HRV indices that would accurately identify the persons with relatively low risk of SCD among the individuals with LVEF ≤35%. To the best of our knowledge, this is the first published study of this type.

Although we were able to distinguish between patients from EVENT_(+) and EVENT_(−) groups based on BRS ≥3 ms/mmHg, the NPV for this parameter (>90%) was not high enough to safely postpone ICD implantation in individuals with LVEF ≤35%. In turn, BRS ≥6 ms/mmHg provided 100% NPV in the prediction of the EVENT during either a 12- or 24-month follow-up, but the increase in the negative predictive value took place at an expense of the number of patients who satisfied this criterion.

Neither the time-domain HRV indices nor LF/HF were useful in the prediction of the EVENT (Tables 3 and 4), which is consistent with the results of previous studies [41, 42]. The only parameter with a prognostic value was LFnu; LFnu ≥23 distinguished accurately between patients from EVENT_(+) and EVENT_(−) groups, but 100% NPV was achieved only for LFnu ≥73; likewise for BRS, setting the cut-off value for LFnu at such high level was reflected by a decrease in the number of patients who satisfied this criterion.

### Prognostic value of MTWA analysed jointly with ANS parameters in the identification of low-arrhythmic risk patients

Due to complex pathomechanism of malignant ventricular arrhythmias, patients with these conditions require comprehensive evaluation based on multiple parameters. Published evidence suggests that the multivariate predictive models may provide more accurate estimates of long-term arrhythmic risk than any single parameter [34, 41–45]. Such approach seems to be particularly justified taking into account the primary objective of this study, i.e. identification of patients with a relatively low risk of arrhythmic events. In our study, such individuals were identified most accurately when the result of MTWA test was analysed jointly with BRS. Our findings imply that patients in whom implantation of ICD can be postponed for up to 12 months due to virtually null risk of malignant ventricular arrhythmia may be selected based on the negative result of MTWA test (Fig 1); furthermore, we showed that the intervention can be delayed by another 12 months if the negative result of MTWA test co-exists with BRS ≥3 ms/mmHg (Fig 2). These findings suggest that joint evaluation of MTWA and BRS may have important practical implications. Also the negative result of MTWA co-existing with LFnu ≥23 provided 100% NPV in the prediction of malignant ventricular arrhythmia, but solely during initial 12 months of the follow-up. Although the negative result of MTWA test and LFnu ≥73 provided 100% NPV during a 24-month-long follow-up, the number of low-risk patients who satisfied those two criteria was markedly lower (Table 6).

### Study limitations

This study has a few potential limitations. A major limitation of this work is that ICDs were implanted at the discretion of the treating physician. The only way to really eliminate this potential source of bias would randomization to ICD implantation (which is difficult to perform in the light of the current guidelines) or implantation all patients with ICDs (in order to reduce this limitation we performed additional sub-analyzes in the ICDs group which is in supporting information (S1 File) which confirm the results from the whole group). Next, the nonrandom allocation of ICDs is particularly problematic because the majority of outcome events in the EVENT (+) group were “appropriate “ICD shocks, which are well-recognized to overestimate the true incidence of SCD. Other our limitation is reporting NPV at 12 months that may be not very useful because the annual event rates in primary prevention ICDs are relatively low, the time course of benefit for primary prevention ICDs takes several years to accrue and the suggestion to repeat MTWA+/- autonomic testing every 12 months could not seem very practical. However, searching for the possibility of identifying patients with minimal risk of arrhythmia, the Authors found that caution and repetitive tests (every 12 months) may be considered as helpful to postpone MTWA implantation especially in the patients with present temporary contraindications or in countries with poor economical situation. Next limitation: over 60% of the cohort had QRS ≥ 120 ms, but only 10% (because the economical situation at the years when the patients were included) had ICD—the results in settings where CRT is more available need to be veryfied. Additionally, our study is singe-centre study, with small sample size and variety endpoints, therefore the presented findings should be considered only as preliminary results.

## Conclusion

Well-known, non-invasive parameters, such as MTWA, BRS and short-term HRV indices may be helpful in the identification of individuals with a relatively low risk of malignant ventricular arrhythmia among patients with ischemic left ventricular systolic dysfunction; in such persons, implantation of ICD could be safely postponed.

## Supporting information

S1 Table Data sets. (XLSX)

S1 File Additional sub-analyzes in the ICDs group. (PDF)
