# Supplementary material for: Microvolt T-wave alternans and autonomic nervous system parameters can be helpful in the identification of low-arrhythmic risk patients with ischemic left ventricular systolic dysfunction
Source: PLoS One. 2018 May 3;13(5):e0196812. doi: 10.1371/journal.pone.0196812 (PMC5933691; doi:10.1371/journal.pone.0196812)
Supplement: S1 File — (PDF) [file pone.0196812.s002.pdf]

Table A. Clinical and demographic characteristics of the patients with ICD and comparison between the EVENT_(+) and EVENT_(-) groups

	**All N=107**	**EVENT_(+) N=29**	**EVENT_(-) N=78**	**p***
Age [years]	64 (58 – 72)	65 (61 – 73)	63 (58 – 70)	0.054
Males, n (%)	96 (90)	24 (83)	72 (92)	0.164
MI history n (%)	96 (90)	24 (83)	72 (92)	0.164
Revascularization, n (%)	97 (91)	25 (86)	72 (92)	0.454
LVEF (%)	30 (24 – 32)	28 (20 – 32)	30 (25 – 35)	0.071
NYHA class				0.553
- NYHA I, n (%)	14 (13)	2 (7)	12 (15)	
- NYHA II, n (%)	70 (65)	20 (69)	50 (64)	
- NYHA III, n (%)	23 (21)	7 (24)	16 (21)	
QRS≥120 ms, n (%)	66 (61)	21 (72)	45 (58)	0.186
VPCs>10/godz., n (%)	52 (49)	16 (55)	36 (46)	0.515
nsVT, n (%)	40 (37)	12 (41)	28 (36)	0.656
MTWA_non-neg, n (%)	78 (73)	27 (93)	51 (38)	**<0.003**
- beta-adrenolytics, n (%)	103 (96)	28 (97)	75 (96)	1.000
- ACE-inhibitor or ARB, n (%)	102 (95)	27 (93)	75 (96)	0.611
- spironolactone, eplerenone, n(%)	58 (54)	13 (45)	45 (58)	0.278
- aspirin, n (%)	96 (90)	24 (83)	72 (92)	0.164
- amiodarone, n (%)	14 (13)	3 (10)	11 (14)	0.754
- statins, n (%)	97 (91)	28 (97)	69 (88)	0.281
- digoxin, n (%)	6 (6)	3 (10)	3 (4)	0.341
-arterial hypertension, n (%)	72 (67)	15 (52)	57 (73)	0.062
- diabetes, n (%)	34 (32)	10 (34)	24 (31)	0.816
Renal function:				0.528
GFR>60 ml/min, n (%)	76 (71)	22 (76)	54 (69)	
GFR 30- 59 ml/min, n (%)	26 (25)	5 (17)	21 (27)	
GFR<30 ml/min, n (%)	5 (5)	2 (7)	3 (4)	
- hypercholesterolaemia, n (%)	76 (71)	20 (69)	56 (72)	0.813
- history of tobacco smoking, n (%)	78 (73)	22 (76)	56 (72)	0.808
CRT-D	14 (13%)	4 (14%)	10 (13%)	1.000

Abbreviations: ACE – angiotensin converting enzyme, ARB – angiotensin receptor blockers; MI – myocardial infarction; LVEF –left ventricular ejection fraction; NYHA – classification according New York Heart Association; VPCs – ventricular premature contractions, nsVT – nonsustained ventricular tachycardia; MTWA_non-neg – positive and indeterminate results for microvolt T-wave alternans; GFR – glomerular filtration ratio; ICD – implantable cardioverter-defibrillator; CRT-D – cardiac resynchronization therapy device with ICD

** p value for comparison between EVENT_(+) and EVENT_(-) groups*

Table B. BRS and HRV parameters in patients with ICD from EVENT_(+) and EVENT_(-) groups

	**EVENT_(+) (n=29)**	**EVENT_(-) (n=78)**	***p**
Mean HP (ms)	1064 (965 – 1169)	1042 (962 – 1169)	0.282
SDNN (ms)	21.35 (14.38 –40.28)	27.10 (17.80 – 34.58)	0.281
RMSSD (ms)	16.20 (10.01 – 35.68)	16.35 (10.01 –35.68)	0.454
pNN50 (%)	0.02 (0 – 12.99)	0.03 (0 – 6.28)	0.385
TP (ms^2)	369.90 (157.48 – 1109.75)	503.25 (157.48 – 1109.75)	0.345
LFnu	30.35 (14.18 –64.08)	57.05 (30.55 – 74.85)	**<0.024**
LF/HF	0.44 (0.17 – 1.78)	1.33 (0.44 – 2.98)	**<0.024**
BRS (ms/mmHg)	2.74 (2.20 – 4.76)	5.09 (2.53 –9.78)	**<0.013**

Abbreviations: HP – heart period; SDNN – standard deviation of the average R-R intervals of the sinus rhythm; RMSSD – square root of the mean squared difference of successive R-R intervals; pNN50 – proportion of successive R-R intervals that differ by more than 50 ms; TP – total power; LFnu – spectral power in low-frequency range (0.04 – 0.15 Hz) expressed in normalized units; LF/HF – LF to HF ratio; BRS – baroreflex sensitivity

** p value for comparison between EVENT_(+) and EVENT_(-) groups*

Table C. Univariate and multivariate Cox models estimating likelihood of the EVENT during the follow-up based on the result of MTWA test, BRS and LFnu indices in patients with ICD

	**Unadjusted**		**Adjusted***	
	p	**Hazard ratio (95% CI)**	**p**	**Hazard ratio (95% CI)**
LVEF (%)	**0.021**	0.93 (0.88 – 0.99)	-	-
MTWA_non-neg	**0.002**	9.13(2.19 – 38.00)	**0.019**	5.69 (1.33 – 24.34)
BRS (ms/mmHg)	**0.018**	0.80 (0.66 – 0.96)	**0.015**	0.79 (0.66 – 0.95)
LFnu	**0.033**	0.98 (0.97 – 1.00)	**0.019**	0.98 (0.96 – 1.00)
LF/HF	0.723	0.97 (0.82 – 1.15)	0.745	0.97 (0.80 – 1.17)

Abbreviations: CI – confidence interval; LVEF – left ventricular ejection fraction; MTWA_non-neg – positive and indeterminate results for microvolt T-wave alternans; BRS – baroreflex sensitivity; LFnu – relative spectral power in LF range, expressed in normalized units; LF/HF – LF to HF ratio

**Adjusted for LVEF*
